# Instant *Candida albicans* Detection Using Ultra-Stable Aptamer Conjugated Gold Nanoparticles

**DOI:** 10.3390/mi15020216

**Published:** 2024-01-31

**Authors:** Kimberley Clack, Mohamed Sallam, Serge Muyldermans, Prabhakaran Sambasivam, Cong Minh Nguyen, Nam-Trung Nguyen

**Affiliations:** 1 Queensland Micro and Nanotechnology Centre (QMNC), Nathan Campus, Griffith University, Nathan, QLD 4111, Australia; kimberley.clack@griffithuni.edu.au (K.C.); mohamed.sallam@griffithuni.edu.au (M.S.); cong.nguyen2@griffithuni.edu.au (C.M.N.); 2 School of Environment and Science (ESC), Nathan Campus, Griffith University, Nathan, QLD 4111, Australia; 3 Griffith Institute for Drug Discovery (GRIDD), Nathan Campus, Griffith University, Nathan, QLD 4111, Australia; 4 Laboratory of Cellular and Molecular Immunology (CMIM), Vrije Universiteit Brussel, 1050 Brussels, Belgium; serge.muyldermans@vub.be; 5 Centre for Planetary Health and Food Security, Nathan Campus, Griffith University, Nathan, QLD 4111, Australia

**Keywords:** bioconjugation, nanotechnology, gold nanoparticles, colorimetric analysis, *Candida albicans*

## Abstract

Fungal pathogens such as *Candida albicans* have significant impacts on women’s health and the economy worldwide. Current detection methods often require access to laboratory facilities that are costly, inconvenient, and slow to access. This often leads to self-diagnosis, self-treatment and eventual antifungal resistance. We have created a rapid (within five minutes), cost-effective, and user-friendly method for the early detection of *Candida albicans*. Our platform utilises aptamer-tagged-gold-core-shell nanoparticles for *Candida albicans* detection based on the presence of 1,3-β-d glucan molecules. Nanoparticle aggregation occurs in the presence of *Candida albicans* fungal cells, causing a redshift in the UV-visible absorbance, turning from pink/purple to blue. This colour change is perceptible by the naked eye and provides a “yes”/“no” result. Our platform was also capable of detecting *Candida albicans* from individual yeast colonies without prior sample processing, dilution or purification. *Candida albicans* yeast cells were detected with our platform at concentrations as low as 5 × 10^5^ cells within a 50 μL sample volume. We believe that this technology has the potential to revolutionise women’s health, enabling women to test for *Candida albicans* accurately and reliably from home. This approach would be advantageous within remote or developing areas.

## 1. Introduction

Vaginal infections are the most prevalent health problem in women, and this is due to incompetent diagnoses, inappropriate treatments, and antibiotic resistance [1]. The financial burden of *Candida* fungal infection is substantial. The treatment for fungal infections such as *Candida albicans* includes antifungal pessaries and single-dose (fluconazole) or two-dose (itraconazole) oral therapy. In 2013, global market was estimated to be US $600 million, including $257 million in sales of the market leader Canesten (clotrimazole; Bayer, Leverkusen, Germany) [2]. *Candida albicans* is the most common cause of both mucosal and systemic infection. This species is responsible for approximately 70% of fungal infections worldwide [3]. Acute vulvovaginal candidiasis (VVC) is one of the most common *Candida* infections and affects 75% of the female population. Up to 10–15% of women develop recurrent infections that are defined as three or more episodes in a twelve-month period [3]. VVC is the second-most common and frequent vaginal infection after bacterial vaginosis [4]. Predisposing factors for VVC include vaginal dysbiosis after antimicrobial therapy, elevated oestrogen levels (caused by pregnancy, hormone-replacement therapy or oral contraceptive use), and uncontrolled diabetes. Recurrent VVC (RVVC) is more severe than recurrent VVC; however, it is less common [5]. Overall, VVC and RVVC severely impact patients’ quality of life and are associated with low self-esteem, mental distress, physical pain and sexual dysfunction [6]. Vaginal symptoms such as odour, discharge and itching are frequent causes of suffering and discomfort in women of reproductive age suffering from VVC [7]. These symptoms not only are an epidemiological and clinical problem but are also a larger psychological and social issue [7]. Furthermore, VVC and RVVC are also implicated in preterm labour, late miscarriage, infertility and pelvic inflammatory disease [6]. Invasive blood-based fungal infection (candidemia) may occur in immunocompromised patients (such as those suffering from HIV, neutropenia or dysbiosis of the microbiota), resulting in patient death [8]. *Candida albicans* infection is problematic within hospitals as the fungus may be present on abiotic surfaces such as implants, prosthetic devices or pacemakers. *Candida albicans* is likely to be transmitted to patients via healthcare professionals [8]. If infected, the patient is often required to undergo additional surgical procedures to remove sources of infection for successful *Candida* management [8].

Therefore, early detection of *Candida albicans* is essential. Traditional detection methods include observing clinical symptoms such as whitish vaginal discharge and itching, microscopy for colony appearance, and as observing pigmentation changes within a chromogenic culture medium [6]. However, these detection methods involve inconvenience and patient suffering. Moreover, mocroscopic examination of these animals lacks accuracy and precision due to the diverse morphologies of vaginal microorganisms and subjectivity [6]. Furthermore, culture-based assessments of the vaginal microbiome are hampered by diverse growth requirements or even the uncultivable nature of various strains [6]. SeptiFast is a commercial molecular-based platform for the diagnosis of candidaemia and invasive candidiasis. The SeptiFast platform can detect up to five *Candida* species with a high sensitivity and specificity [9]. The test is performed directly on a sample of blood; however, extraction of both human and pathogen DNA involves mechanical lysis and manual spin columns, requiring a contamination-controlled workflow [9]. The extracted DNA is then amplified via a real-time PCR assay, and the amplicons are hybridised to fungal species-specific fluorescent probes for pathogen identification. However, the test is lengthy, taking six hours to complete [9]. Other molecular-based diagnostic approaches exist for qualitative and/or quantitative detection of *Candida* species-specific DNA, such as nested and seminested PCR, PCR-enzyme immunoassays, variations of real-time PCR and multiplex PCR followed by DNA sequencing or pyrosequencing [9]. However, the detection of nucleic acids in body fluids is challenging due to the low pathogen count and tough fungal cell walls, which hamper lysis and DNA release. These issues often lead to false-negative results [9]. Furthermore, clinical isolate sequencing is costly, time-consuming, and not yet standardised [6]. PCR inhibitors may also lead to false-negative results. False positive results may also arise due to airborne contamination of specimens, particularly when using panfungal primers that target highly conserved rRNA or other genes [9]. Glucans are the most abundant polysaccharides in fungal cell walls, and they offer an alternative detection approach to amplification-based methods [10]. β-1,3-d-glucan molecules constitute 84% of fungal glucan molecules [11]. The Fungitell platform (Cape Cod, MA, USA) was used to detect *Candida albicans* fungal samples (from serum) via the detection of 1,3-β-d (BDG) glucan molecules. It is the only Food and Drug Administration-approved commercially available BDG detection platform [9]. However, false-positive results may occur due to conditions such as abdominal surgery, haemodialysis, treatment with β-lactam antibiotics and concomitant presence of lipopolysaccharides due to gram-negative bacteraemia [9]. These factors make it difficult to use this platform in the clinical setting. Overall, critical challenges in managing *Candida* fungal infections include early prevention, early detection and rapid initiation of appropriate antifungal therapies [8]. Poorer clinical outcomes are associated with untimely treatment. Furthermore, antifungal resistance presents a new global burden [8]. Therefore, it is essential to design portable, sensitive, rapid, real-time, low-cost point-of-care microbial detection tools [4].

Colorimetric sensing strategies are of great interest as diagnostic tools because they are generally simple and practical. They provide a way to detect a target analyte or biomolecule via a naked-eye-detectable colour change without the need for complex data generation [12]. Specifically, conventional colorimetric platforms for fungal pathogen detection may include (but are not limited to) loop-mediated isothermal amplification (LAMP) for detecting the presence of amplified target fungal DNA [13,14]; HRP-mediated TMB oxidation (horseradish peroxidase tetramethylbenzidine) assays [15] and surface plasmon resonance-based techniques [16]. However, these techniques may require heating equipment (for LAMP), numerous manual handling steps and laboratory access. This approach greatly reduces the likelihood of producing a low-cost, rapid, and portable detection platform. Nanomaterials are commonly used within biosensors as simple alternatives to conventional platforms. Nanomaterials enhance platform sensitivity and lower detection limits by increasing the surface area on which a biorecognition element (such as an aptamer) can be immobilised [17]. The nanomaterials commonly used in biosensing include quantum dots, carbon nanotubes, graphene, polymeric nanomaterials, and metal and metal oxide nanoparticles [17]. Gold nanoparticles are ideal for colorimetric sensing applications due to their unique features, including controllable size, facile synthesis, well-established surface chemistry, catalytic activity, and well-established synthesis and surface plasmon resonance-enhanced optical properties [18]. Gold nanoparticles have been previously used within colorimetric sensing platforms for fungal spore detection. However, despite the merits of these platforms, they may still utilise potentially expensive and specialised equipment for colour analysis, such as a light source and camera setup [19] or a smartphone for colour change pattern analysis [18]. This equipment requirement reduces the portability of the platform and reduces the chance of widespread adoption in resource-limited or financially poor settings. While certain platforms offer distinct colorimetric results without the need for extraneous instrumentation or prior sample preparation, they may suffer in terms of specificity for differentiating between fungal species [20]. Gold nanoparticles have been incorporated into lateral flow platforms for specific fungal pathogen detection. However, these methods required integration with prior universal primer-mediated asymmetric PCR, which requires expensive equipment and access to a laboratory [21].

To address these questions and needs, we developed a rapid, naked-eye-based colorimetric method for *Candida albicans* detection via thiolated (3′ end) aptamer-conjugated gold nanoparticles. The greatest advantage of aptamers over antibody-based platforms is that aptamers can specifically bind to target biomolecules, such as β-1,3-d-glucans, with high affinity and specificity, as well as low immunogenicity and low toxicity [22,23]. Aptamers are also beneficial as target capture agents due to their relative ease of chemical synthesis, tuneable backbone modification and excellent stability over a range of ionic, pH and temperature conditions [23]. Upon receptor–target-directed aggregation, a change in the optical properties of the particles occurs—i.e., a redshift in surface plasmons or a red-to-blue colour change as detected by the naked eye [24,25].

The sodium citrate reduction (of gold chloride) method is a common approach for gold nanoparticle synthesis and was reported as early as 1951 by Turkevich and colleagues [26], with many works following this approach with slight modifications [27,28]. The method involves heating chlorauric acid solution to the boiling point before adding sodium citrate solution under magnetic stirring [26]. By decreasing the amount of citrate, the mean particle diameter can increase and therefore be controlled. This citrate reduction method rapidly results in the formation of gold sols within a few minutes of boiling [26]. The presence of citrate molecules on the surface of gold nanoparticles serves two important functions: First,, the presence of citrate molecules lowers the surface energy of highly active gold nanoparticles, which achieves chemical stability. Secon, the agglomeration of gold nanoparticles is physically stabilised by preventing their agglomeration, which places the nanoparticles in a monodispersed state, and is crucial for their interaction with other molecules [27]. However, citrate capping also imparts a strong negative charge to the surface of gold nanoparticles, attracting cationic molecules through charge-induced electrostatic forces [27]. When binding occurs, overall neutralisation of the charges on the surface of the nanoparticles occurs, decreasing the inter-particle distance, which in turn causes nanoparticle aggregation [27]. As positively charged compounds containing amino groups may be present in human biological fluids; this may interfere with Au-S bonds, which may be unsuitable for our thiolated aptamer-based platform without extensive optimisation steps [29]. The strong and compact adsorption of non-ionic surfactants (such as polyoxyethylene (20) sorbitan monolaurate (Tween-20)) to gold nanoparticles can efficiently suppress the nonspecific surface adsorption of macromolecules such as proteins and DNA [30]. Tween-20 may be incorporated onto the citrate-capped gold nanoparticle surface to prevent the aggregation of gold nanoparticles by shielding the citrate ions [31]. Tween-20 incorporation is a viable protection strategy for assisting the chemisorption of alkanethiols to nanoparticles [31]. This method produces functional peptide nucleic acid-conjugated gold nanoparticles [31].

For our method, we avoided citrate-based synthesis altogether. This avoidance of the heating step (observed in citrate-based synthesis) also allowed us to combine our thiolated aptamers during the nanoparticle formation at room temperature for ease of synthesis within one reaction vial. Notably, we also incorporated glucose, which acts as both a capping agent and a reducing agent, to further enhance our synthesis. While glucose is a well-known reducing sugar, it has a limited reduction ability at ambient temperature. However, glucose can effectively reduce Au^3+^ ions into Au^0^ upon the addition of a small amount of aqueous NaOH [32]. Our method is minimally invasive and can detect *Candida albicans* cells based on the presence of 1,3-β-d glucan molecules in a vaginal fluid simulant solution. Our method does not require blood or plasma or costly or cumbersome laboratory equipment. Our method can detect as few as 5 × 10^5^ *Candida albicans* cells in a volume of less than 50 μL of sample solution. Our method offers an easily perceptible “blue” colour change for a positive result and a “pink” colour change for a negative result. To the best of our knowledge, no specific aptamer-based colorimetric gold nanoparticle-based platform exists for the direct detection of *C. albicans* in vaginal fluid.

## 2. Materials and Methods

### 2.1. Aptamer Selection

The AD1 aptamer k_d_ = 79.76 nM was selected based on prior works. Hou’s group used AD1 to detect different morphological forms of *Candida albicans*, including yeast cells, germ tubes and hyphae, as well as extracellular matrix material [11]. Tang et al. [22] also demonstrated the detection of β-1,3-d-glucan molecules with this aptamer. The AD1 aptamer was thiolated at the 3′ end for binding to gold nanoparticles. A ten-nucleotide length of adenylation site was also incorporated as a spacer.

The sequence is as follows:

5′GCGGAATTCGAACAGTCCGAGCCCACACGTGTGAGAAGGGTGTTATCATGTATTTCGTGTTCCTTTCGTCATTCCTTTGTCTGGGGTCAATGCGTCATAGGATCCCGCAAAAAAAAAA-3′Thiol Modifier C3 S-S.

### 2.2. Vaginal Fluid Simulant

Every day, women produce approximately six grams of vaginal fluid, and at any given time, 0.5–0.75 g of fluid is present [6]. Vaginal fluid is mainly composed of proteins, salts, fatty acids and carbohydrates [6]. Notably, there is a large inter and intraindividual variation in these components; however, for our work, a vaginal fluid simulant solution was prepared from the recipe used by Owen and Katz (1999) [33]. The mixture consisted of NaCl (M.Wt. 58.44), KOH (M.Wt. 56.11), Ca(OH)_2_ (M.Wt. 74.09), bovine Serum albumin, lactic acid (L+) (M.Wt. 90.08), glacial acetic acid (M.W.t. 60.05), glycerol (M.Wt. 92.09), urea (M.Wt. 60.06) and d-glucose monohydrate (M.Wt. 198.17). The combined reagents were diluted to a total reaction volume of 1 L with Milli-Q water. The solution was adjusted to pH 4.2 with HCl, vacuum filtered, and UV sterilised before being aliquoted for later use. This pH adjustment is in accordance with the pH of the average healthy vagina, which is approximately 4 ± 0.5. However, this pH value differs across ethnicities and geographical locations [6].

### 2.3. Fungal Culture and Reagents

*Candida albicans* as cultured on yeast extract peptone dextrose (YEPD) plates that were created according to the following recipe: 5 g of yeast was dissolved in 250 mL of Milli-Q water. Ten grams of peptone (M.Wt 244.33) was added, as was 10 g of glucose (180.16), followed by 10 g of agar. The solution was heated to combine the reagents, and Milli-Q water was added to the 500 mL mark. The solution was autoclaved and stored at 4 °C overnight, after which the plate was poured the following day. *Candida albicans* strains were cultured by adding a portion of lyophilised stock to 400 μL of Miller′s LB broth solution and covering YEPD plates with the fungal solution to ensure colony distribution. The plates were incubated at 30 °C. After five days of growth, the fungal colonies were harvested for experiments. The cells were resuspended in a vaginal simulant solution to achieve an OD_600_ = 1.0 for OD analysis. *Botrytis cinerea* was cultured on V8 agar plates that were created according to the following procedure: 8 g of bacteriological agar, 1.5 g of CaCO_3_ (M.Wt 100.09) (previously dissolved in 50 mL of Milli-Q water) and 90 mL of V8 vegetable juice were combined, and Milli-Q water was added to the 400 mL mark. The solution was vacuum-filtered, autoclaved, and stored overnight (at 4 °C). The mixture was heated and the plate was poured the following day. *Botrytis cinerea* was obtained in-house and cultured by slicing a portion of agar from the stock fungal plate and placing it on a fresh plate incubated at room temperature. Spores were harvested by scrubbing the plates with pipette tips after five days of growth.

### 2.4. Gold Nanoparticle Synthesis

The chemicals used for our instant oligo-functionalised nanoparticles included auric-chloride (gold (III) chloride trihydrate tetrachloroauric acid (HAuCl_4_·3H_2_O), M.Wt. 393.83 and tween-20 (polyethylene glycol sorbitan monolaurate) which were purchased from Merc (Hackensack, NJ, USA). Glucose (M.Wt. 180.16) and NaOH (M.Wt. 40) were also used as part of the nanoparticle synthesis. Aptamers (thiolated single-stranded DNA anti-(1,3)-β-d-glucan (ssDNA-SH)) were designed with online software tools and were purchased from Integrated DNA Technologies (IDT) (Coralville, IA, USA). Milli-Q water (≥18 MΩ·cm) was used for all reagent preparations. Our gold nanoparticles were prepared in a single glass vial within five minutes according to the following protocol: 440 μL of 0.1% Tween-20 H_2_O was added to the glass vial, followed by 60 μL of 10 mM HAuCl_4_·3H_2_O solution. Then, 250 μL of 100 mM glucose was added, followed by the subsequent addition of 250 μL of 50 nM thiolated single-stranded DNA (anti-BDG) aptamer. Aptamers were previously prepared from the stock solution by mixing 1.25 μL of 100 μM aptamer stock solution with 1250 μL of 0.01% Tween-20 H_2_O solution. The glass vial containing all reagents was then magnetically stirred for 5 min at room temperature (250 RPM). Finally, 25 μL of Sodium hydroxide (1 M) reducing agent was added to the glass vial to complete the synthesis process of our stable gold nanoparticles. A combination of thiolated aptamers and Tween-20 solution enhanced nanoparticle stability. Tween-20 servs as a capping agent and surfactant that causes the anionic nanoparticles to form and interact better with the aqueous environment and to remain in solution [34]. Maintaining the correct nanoparticle size was highly dependent on the Tween-20 concentration [34]. At larger Tween-20 concentrations, significant blueshifts can occur [34]. In general, nanoparticles may continue to grow for 24 h after synthesis, and those with less stable capping agents may continue to grow slowly after 24 h [34]. To ensure reproducibility and a smaller size, our nanoparticles were used within an hour of synthesis. The aptamer concentration is also critical to nanoparticle performance and was carefully selected. While a high aptamer concentration increases the stability of gold nanoparticles, it may reduce nanoparticle sensitivity due to residual aptamers, or may cause background interference [35]. Notably, our particles were created in high-yield batches that were capable of being upscaled to produce 10 mL volumes if needed. Our particles did not require sonication, centrifugation, or concentration and could be used directly after synthesis for immediate sample testing. We believe that this increase in viscosity of the concentrated nanoparticle solution also aids in reducing nonspecific aggregation.

### 2.5. Nanoparticle Characterization

**Figure 1:**
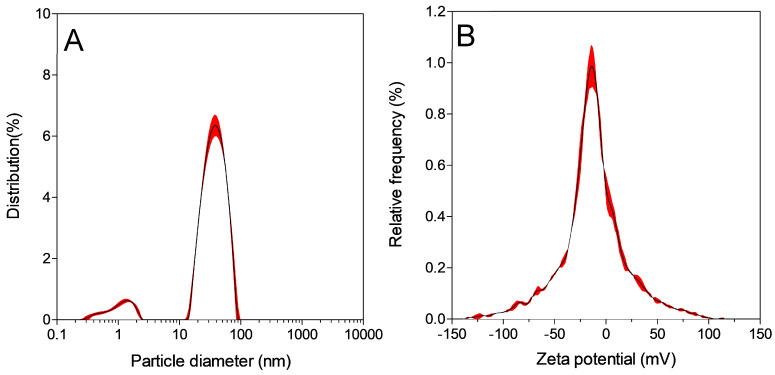

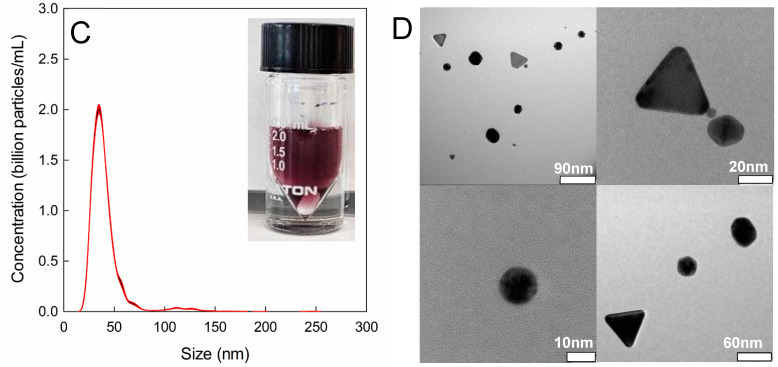
The hydrodynamic diameter (62.1 nm) (**A**) of the gold nanoparticles. (**B**) shows a mean particle zeta potential of −16.82 mV. (**C**) shows a mean particle size of 41.4 nm (±0.5 nm), while the particle mode was 35.5 nm (±0.5 nm) with a standard deviation of 19.3 nm (±1.5 nm). The Inset of (**C**) shows the colour of the formed nanoparticles (peak UV-visible absorbance at 528 nm). (**D**) High-resolution TEM images showing the different gold nanoparticle shapes, including spheresand triangles.

Nanoparticles were characterised by several techniques. UV-visible spectroscopy was carried out on a CLARIOstar microplate reader (BMG LABTECH, GmbH, Ortenberg, Germany). The surface plasmon resonance was clearly visible as a peak at the 528 nm position. This peak is within the range of 520 to 580 nm, which is consistent with gold nanoparticle formation, as per the literature [20]. The resultingnanoparticles are shown in Figure 1C (inset). Particle size and concentration analyses were performed using a dynamic light scattering (DLS) particle size analyser—LiteSizer (Anton Paar, Graz, Austria) machine and a nanoparticle tracking analyser, with a NanoSight NS300 (Malvern Panalytical, Malvern, UK) instrument. Additionally structure characterisation was performed via high-resolution transmission electron microscopy (TEM) (JEOL 2100, 200 kV TEM, Tokyo, Japan). pH analysis was carried out via pH indicator strips (LabCo, Loxstedt-Stotel, Germany).

### 2.6. pH and Temperature Dependence

The aggregation of nanoparticles involves the primary reaction between the aptamers and *Candida albicans* glucan molecules, and the secondary reaction involves the aggregation of the nanoparticles to form aggregates. Therefore, the rate of aggregation is expected to be highly pH-dependent [36]. The pH of the stock solution of synthesised nanoparticles was tested with a pH indicator strip (LabCo Germany). The particle pH was determined to be 11. The viscosity of the vaginal fluid simulant solution was 4. When combined with vaginal fluid simulant, *C. albicans* remained at pH 4. *Botrytis cinerea* also remained at pH 4. This indicated that nanoparticle aggregation was not the result of a pH change alone. Importantly, the pH and composition of the vaginal fluid simulant solution did not cause the nanoparticles to aggregate non-specifically, which is essential for future clinical applications. Typically, chemical reaction rates usually increase with increasing temperature. This is the opposite case for nanoparticle-based aggregation, as the aggregation rate increases with decreasing temperature [36]. This is due to the aggregation reaction involving a negative change in entropy. Therefore, increasing the temperature results in an increase in free energy, which decreases the stability of the formed aggregates [36]. For example, the rate of aggregation at 20 °C is 2.5 times greater than the rate of aggregation at 37 °C [36]. This implies that temperatures closer to room temperature were suitable for the synthesis of our nanoparticles. Consequently, a result can occur without the need for heating apparatuses. Furthermore, cooling was not needed to achieve our rapid results.

### 2.7. Hydrodynamic Particle Size and Zeta Potential Measurement Using Dynamic Light Scattering (DLS)

DLS was performed on an Anton Paar LiteSizer DLS Particle Size Analyser machine. DLS was used to characterise the hydrodynamic size and surface charge of the synthesised gold nanoparticles. To analyse the particle size, 1 mL of nanoparticle solution was placed into a 10 × 10 × 45 mm disposable cuvette that was placed in the LiteSizer machine, and the results were recorded. The hydrodynamic diameter was 62.1 nm. For eta potential analysis, 1 mL of the same particle solution was placed into an omega cuvette and zeta potential was measured. With these techniques, we were able to accurately calculate the hydrodynamic size and surface charge of our nanoparticles. The particles were anionic with a mean zeta potential of −16.8 mV and a distribution peak of −13.3 mV ± 1.24. The electrophoretic particle mobility was −1.31 µm.cm/V.s. The conductivity was 3.41 mS/cm ± 0.04. The polydispersity index was 24.54%. All the individual measurements were an average of three replicates. The results are shown in Figure 1A,B.

### 2.8. Mean Particle Size and Concentration with Nanoparticle Tracking Analysis (NTA)

NTA was performed on a NanoSight NS300 (Malvern Panalytical, Malvern, UK) device to discern the size of the nanoparticles that were undergoing Brownian motion. For these measurements, 1 mL of nanoparticle solution was transferred to a 1 mL syringe that was connected to the pump of the device. The particle movement and dispersal of light were recorded by a camera at 25 °C via a blue laser. The mean particle size was 41.4 nm (±0.5 nm), while the particle mode was 35.5 nm (±0.5 nm) with a standard deviation of 19.3 nm (±1.5 nm). The results are shown in Figure 1C).

### 2.9. Particle Shape Analysis viaTransmission Electron Microscopy (TEM)

Transmission electron microscopy (TEM) (JEOL 2100 operating at 200 kV) was also used to further characterise the size and shape of the gold nanoparticles. This method enabled us to accurately determine the average size of the nanoparticles, as well as assess their uniformity and any potential morphological variations. We observed interesting crystallographic orientations of the particles, consisting mainly of triangles. The results are shown in Figure 1D).

### 2.10. Selection of Reagents/Fungal Samples for Platform Specificity Testing

Dextran ([C_6_H_10_O_5_]_n_), starch ([C_6_H_10_O_5_]_n_) and *Botrytis cinerea* were chosen for testing the stability and specificity of the nanoparticles within other glucan solutions. These reagents were selected due to their availability and glucan composition. Dextran is a complex glucan that is composed of a main chain of d-glucose linked by α-(1–6) bonds with possible d-glucose branches with α-(1–4), α-(1–3), or α-(1–2) bonds [37]. Dextran exhibits variation in terms of its branching and molecular weight based on temperature or sucrose concentration [37]. Dextran is highly water soluble [38], and it is expected that the glucan molecules will be highly amenable to exposure to the AD1 aptamers of nanoparticles—which is essential for nanoparticle specificity testing. The use of dextran as a control sample was also clinically relevant to this work as dextran may be isolated from different sources of lactic acid bacteria that are found in vaginal swabs. These bacteria include *Lactobacillus gasseri* and *Lactobacillus acidophilus* [37].

Starch granules are composed of alpha glucan (amylose and amylopectin) polysaccharides that represent 98–99% of the dry weight [39]. Amylose is a linear glucan that contains α-(1–4) and α-(1–6) linkages. The linkage size and structure differ depending on biological origin [39]. Amylopectin contains a heavily branched structure that is built from 95% α-(1–4) and 5% α-(1–6) linkages [39]. A soluble version of starch was used for the purpose of our experiments.

Both dextran and starch were diluted in deionised water to give respective concentrations of 50 mg/mL. This procedure was used to ensure a high concentration of glucan molecules for each of the reagents.

*Botrytis cinerea* is a necrotrophic plant pathogen that colonises plant tissues, causes gray mold in vegetables, and causes softening in fruits [40]. The hyphae of *Botrytis cinerea* penetrate plant tissues through natural openings or wounds. This fungus causes significant economic losses at the pre-or postharvest stages, and it impacts a broad range of crops, including tomato (*Solanum lycopersicum*), potato (*Solanum tuberosum*), grapes (*Vitis vinifera*), and strawberry (*Fragaria* spp.) [40]. *Botrytis cinerea* has been shown to produce an exopolysaccharide known as β-(1,3)-(1,6)-d-glucan [40]. The presence of these glucans in the extracellular matrix of *Botrytis cinerea* is well documented [41]. As such, excess *Botrytis cinerea* mycelia (50 mg) were harvested and sonicated for addition to nanoparticle solutions. *Botrytis cinerea* was chosen as a readily available control due to overexpression of β-(1,3)-d-glucans. The use of *Botrytis cinerea* was useful for testing the specificity of our AD1 aptamer as our aptamer is designed to recognise the specific microenvironment of *Candida albicans* BDG only.

### 2.11. Image Processing

All of the images used in this study were photographed, and the images were cropped to the same size. The image brightness was increased by 40% for all the results for visual clarity. The images were subsequently converted to red channel image components through ImageJ software (version 1.54 g Java 1.8.0_345) (National Institutes of Health, New York, NY, USA), and the brightness intensity was measured across all of the sample images. This red-channel analysis served to differentiate blank (pink) solutions from positive (blue) results. As the nanoparticles aggregated due to the presence of *Candida albicans* fungal targets, the solution turned blue, and the brightness intensity of the red pixels decreased.

## 3. Results and Discussion

**Figure 2:**
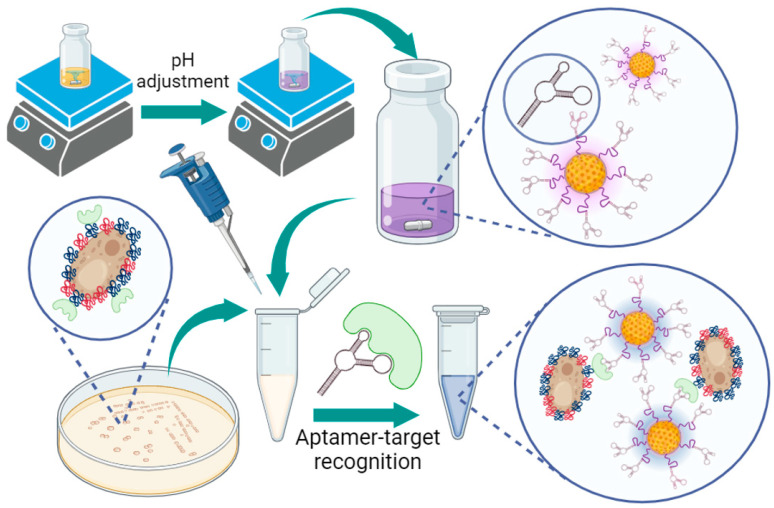
Schematic representation of the synthesis of aptamer-tagged-gold-core-shell nanoparticles for colourimetric aggregation-based detection of *Candida albicans*. Tween-20 in H_2_O, HAuCl_4_·3H_2_O, glucose and selective *Candida albicans* aptamers were combined. The solution was magnetically stirred for five minutes before pH adjustment. After pH adjustment, nanoparticles are promptly formed. Adding the nanoparticles to vaginal fluid simulant containing *C. albicans* causes a redshift in the UV-visible absorbance (blue colour) based on nanoparticle aggregation. Aggregation occurs after recognition of the specific aptamer-target 1,3-β-d glucan molecule. The scheme is illustrated and reproduced with permission from BioRender.

The assay principle is schematically shown in Figure 2. The synthesis of aptamer-tagged-gold-core-shell nanoparticles begins with the stepwise addition of Tween, HAuCl_4_·3H_2_O, glucose and the AD1 aptamer (for specific binding to 1,3-β-d glucan molecules). After magnetic stirring for five minutes, the NaOH-reducing agent was added to complete the formation of gold nanoparticles that were anionic in charge. In this manner, the nanoparticles are stabilised by charge repulsion. The synthesised nanoparticles were then ready for testing with a vaginal fluid simulant containing spiked *C. albicans* fungal samples. In the presence of *Candida albicans*, nanoparticles aggregate based on charge changes. Nanoparticle aggregation results in a redshift (or blue colour change) on the plasmonic spectrum [42].

### 3.1. Proof-of-Concept and Specificity Testing

**Figure 3:**
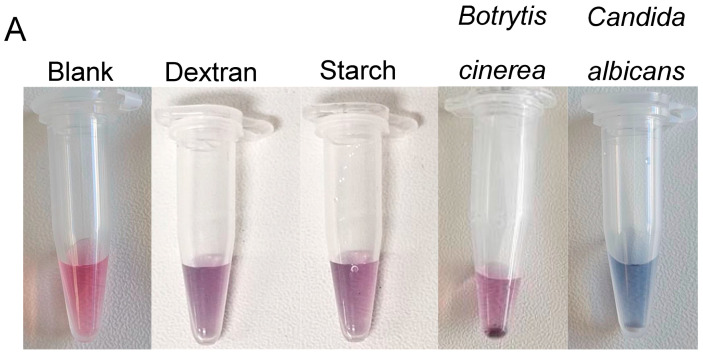

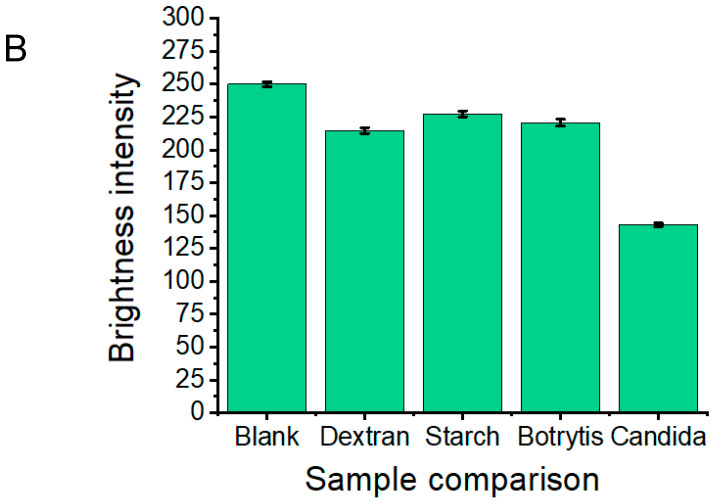
Colorimetric results (**A**) indicating that the nanoparticles were specific for the detection of *Candida albicans* (blue) in comparison to dextran, starch and *Botrytis cinerea* samples which were relatively aggregation-free (pinkish purple). All the results are comparable to those of the blank solution which was pink. (**B**) Red channel brightness intensity measurements for the comparison of blank, nonspecific samples (dextran, starch and *Botrytis cinerea*) and *Candida albicans* samples. The nonspecific samples more closely resembled the blank sample red channel brightness intensity. *Candida albicans* wasdistinctly different from both the blank and nonspecific samples, as *Candida albicans* displayed a lower level of red channel brightness intensity than the other samples.

Specificity testing was performed to determine whether polysaccharides containing branched α linked glucans or an abundance of BDG (from *Botrytis cinerea*) would interfere with the nonspecific aggregation of the nanoparticles. Then, 50 μL of 50 mg/mL dextran solution was added to 50 μL of undiluted nanoparticle solution. Then, 50 μL of 50 mg/mL starch solution was added to 50 μL of undiluted nanoparticle solution. An excess of *Botrytis cinerea* mycelium (50 mg) was added to the vaginal fluid-mimicking solution with 50 μL of nanoparticle solution. The results were compared to those of the blank solution containing 50 μL of vaginal fluid simulant solution and 50 μL of nanoparticle solution. A positive test for the detection of one *Candida albicans* colony is also shown for reference. The results showed that compared to those in the blank solution, only minimal nonspecific aggregation occurred in both the dextran and Starch tests. Only minimal nanoparticle aggregation was present within the sample containing *Botrytis cinerea* mycelia. Notably, the presence of *Candida albicans* caused a “blue” colour change due to nanoparticle aggregation based on aptamer recognition of the presence of 1,3-β-d glucan molecules. This “blue” colour is distinct from that of the nonspecific targets (dextran, starch and *Botrytis*) and the blank solution, demonstrating the specific detection of one colony of *C. albicans* based on the specific aptamer recognition of *C. albicans* 1,3-β-d glucan molecules. Colorimetric results were visible for each sample within 5 min of adding the nanoparticles. Red channel brightness intensity analysis revealed that the nontargets (dextran, starch and *Botrytis cinerea*) were comparable to the blank solution. Moreover, the red channel brightness intensity significantly decreased for the positive detection of *C. albicans*. The results are shown in Figure 3.

### 3.2. SemiQuantitative Candida albicans Yeast Cell Detection via OD_600_

**Figure 4:**
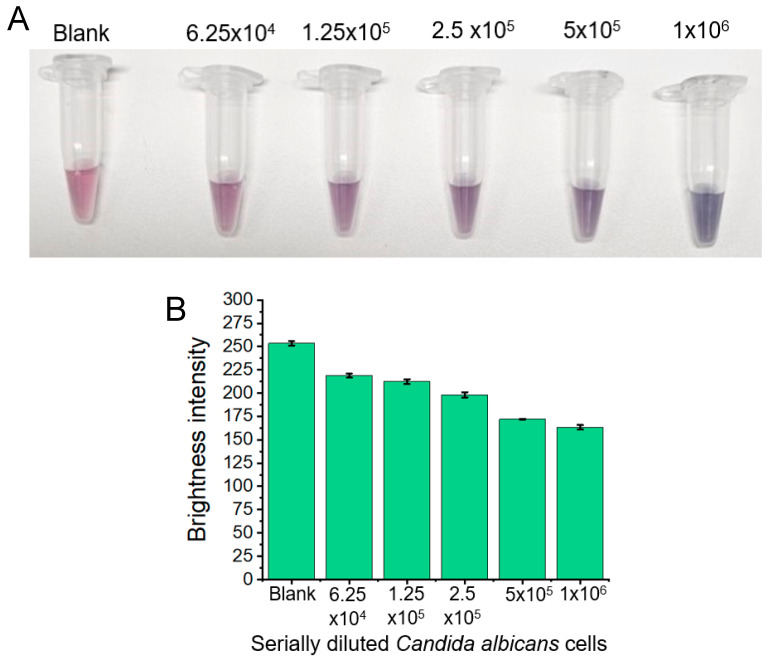
(**A**) shows serially diluted samples of *Candida albicans* fungal cells. Serial dilutions ranged from 1 × 10^6^ cells (blue) to 6.25 × 10^4^ cells (pink). As the concentration of *Candida albicans* fungal cells decreased, each solution more closely resembled the pink blank solution. (**B**) Images of the serially diluted *Candida albicans* fungal samples. As the concentration of *Candida albicans* fungal cells decreased, so did the red channel brightness intensity. Conversely, as the concentration of *Candida albicans* cells increased, the solution turned more “blue” due to increased nanoparticle aggregation and, therefore, a decrease in the red channel brightness intensity was observed.

Semiquantitative analysis of *Candida albicans* yeast was performed on serially diluted fungal samples (Figure 4). Initially, a portion of a *Candida* albicans colony was spiked into a vaginal fluid simulant to achieve an optical density (OD) reading of 1.0 at 600 nm. This OD_600_ value served as an approximation of the number of *Candida albicans* fungal cells in the solution. At OD_600_ = 0.5, an approximate fungal cell concentration of 1 × 10^7^ cells/mL is expected [43]. Therefore, in the initial 1 mL cell solution, approximately 2 × 10^7^ cells were present. Fifty microlitres of these cells were combined with 50 μL of nanoparticles, and the results were observed. From right to left, serial dilutions included 1 × 10^6^, 5 × 10^5^, 2.5 × 10^5^, 1.25 × 10^5^, and 6.25 × 10^4^ cells, with the blank on the left. Each concentration contained 50 μL of spiked fungal cells and 50 μL of nanoparticle solution. The colorimetric results indicated a “blue” colour change due to the aggregated nanoparticles; therefore, *Candida albicans* was detected 1 × 10^6^ and possibly 5 × 10^5^ *Candida albicans* cells (turning slightly blue) within a 50 μL sample. Red channel brightness intensities were also measured for each of the results and are presented below. The results showed a clear correlation between decreasing red channel brightness intensity and increasing concentrations of *Candida albicans* fungal cells.

## 4. Conclusions and Future Direction

Fungal pathogens such as *Candida albicans* have a significant impact on women’s health and the economy globally. Current detection methods often require access to laboratory facilities that are costly, inconvenient, and slow to access. This often leads to women self diagnosis, self treatment and eventual antifungal resistance. Therefore, there is a special need to create a rapid, cost-effective, sensitive and specific *Candida albicans* detection platform that women can use within the comfort of their homes. To achieve this goal, we synthesised a stable type of monodisperse and homogeneous thiolated aptamer-conjugated gold nanoparticle. Our nanoparticles showed a distinctive “blue” colour, which indicates a positive test for *Candida albicans*. The nanoparticle synthesis was simple and required only five reagents. After adding Tween-20 in H_2_O, HAuCl_4_·3H_2_O, glucose and selective *Candida albicans* aptamers, nanoparticles were promptly formed after five minutes of magnetic stirring and NaOH addition. *Candida albicans* detection was based on the presence of fungal β-1,3-d-glucans that were specifically recognised by our thiolated AD1 aptamers. Adding the nanoparticles to vaginal fluid simulant containing *C. albicans* causes a redshift in the UV-visible absorbance (blue colour) based on nanoparticle aggregation. Our nanoparticles could be used to detect *Candida albicans* fungal yeast in spiked samples from vaginal fluid-mimicking solution within five minutes. As few as 5 × 10^5^ yeast cells were detected within a 50 μL sample volume after the addition of 50 μL of nanoparticles. Furthermore, our nanoparticles did not yield a false-positive result when tested with high concentrations of other nonspecific dextran and starch polysaccharides. Additionally, the nanoparticles showed high specificity when they were tested within samples of *Botrytis cinerea* containing excess β-1,3-d-glucans. The merits of our method include rapid nanoparticle synthesis (less than 5 min) from five reagents without additional nanoparticle purification steps. After synthesis, our concentrated nanoparticles were ready for immediate addition to *Candida albicans* fungal samples that were spiked into a vaginal fluid simulant solution. Importantly, *Candida albicans* fungal samples can be used directly with nanoparticles without any purification steps. In future works, nanoparticle synthesis will be optimised to increase nanoparticle stability and yield and to extend shelf life. The clinical relevance of these nanoparticles will be thoroughly investigated by testing them in both healthy patients and patients suffering from *Candida albicans* infections. Furthermore, the ability of the platform to distinguish between similar Candida species will also be investigated. We anticipate that the work performed here will serve as a proof of concept for incorporating gold nanoparticle aptamer-based aggregation technology into a portable and wearable device for *Candida albicans* detection. We believe that our platform will contribute to revolutionising women’s health, by providing women with control over their health rather than relying on clinical tests or preemptive self treatmentwith antifungal medication.

## Data Availability

Data are contained within the article.
